# Why women are not small men: sex-related differences in perioperative cardiopulmonary exercise testing

**DOI:** 10.1186/s13741-020-00148-2

**Published:** 2020-06-04

**Authors:** G. Thomas, M. A. West, M. Browning, G. Minto, M. Swart, K. Richardson, L. McGarrity, S. Jack, M. P. W. Grocott, D. Z. H. Levett

**Affiliations:** 1 Department of Intensive Care, Spaarne Hospital, Haarlem, The Netherlands; 2 Academic Unit of Cancer Sciences, Faculty of Medicine, University of Southampton, Southampton, UK; 3 Anaesthesia Perioperative and Critical Care Research Group, NIHR Biomedical Research Centre, University Hospital Southampton NHS Foundation Trust/University of Southampton, Southampton, UK; 4 Department of Anaesthesia, Maidstone and Tunbridge Wells NHS Trust, Hermitage Lane, Maidstone, Kent, UK; 5 Directorate of Anaesthesia, Derriford Hospital, 9th Floor Terence Lewis Building, Plymouth, UK; 6 Peninsula Schools of Medicine and Dentistry, Plymouth University, Plymouth, UK; 7 Department of Anaesthesia and Critical Care Medicine, Torbay Hospital, Torquay, UK; 8 STRAPH Research Group, School of Sport and Exercise Sciences, University of Kent, Canterbury, UK; 9 Anaesthesia and Intensive Care Medicine, Medway Maritime Hospital, Gillingham, UK; 10 Department of Anaesthesia, University Hospital Crosshouse, Kilmarnock, East Ayrshire, Scotland, UK; 11 Integrative Physiology and Critical Illness Group, Clinical and Experimental Sciences, Sir Henry Wellcome Laboratories, Faculty of Medicine, University of Southampton, University Hospital Southampton NHS Foundation Trust, Mailpoint 810, Tremona Road, Southampton, SO16 6YD UK; 12 Anaesthesia and Critical Care Research Unit, University Hospital Southampton NHS Foundation Trust, Mailpoint 27, D Level, Centre Block, Tremona Road, Southampton, SO16 6YD UK

**Keywords:** Cardiopulmonary exercise testing, Preoperative assessment, Risk prediction, Sex analysis, Gender analysis, Sex characteristics

## Abstract

**Background:**

The use of preoperative cardiopulmonary exercise testing (CPET) to evaluate the risk of adverse perioperative outcomes is increasingly prevalent. CPET-derived information enables personalised perioperative care and enhances shared decision-making. Sex-related differences in physical fitness are reported in non-perioperative literature. However, little attention has been paid to sex-related differences in the context of perioperative CPET.

**Aim:**

We explored differences in the physical fitness variables reported in a recently published multi-centre study investigating CPET before colorectal surgery. We also report the inclusion rate of females in published perioperative CPET cohorts that are shaping guidelines and clinical practice.

**Methods:**

We performed a post hoc analysis of the trial data of 703 patients who underwent CPET prior to major elective colorectal surgery. We also summarised the female inclusion rate in peer-reviewed published reports of perioperative CPET.

**Results:**

Fitness assessed using commonly used perioperative CPET variables—oxygen consumption at anaerobic threshold (AT) and peak exercise—was significantly higher in males than in females both before and after correction for body weight. In studies contributing to the development of perioperative CPET, 68.5% of the participants were male.

**Conclusion:**

To our knowledge, this is the first study to describe differences between males and females in CPET variables used in a perioperative setting. Furthermore, there is a substantial difference between the inclusion rates of males and females in this field. These findings require validation in larger cohorts and may have significant implications for both sexes in the application of CPET in the perioperative setting.

## Introduction

The use of clinical exercise testing is increasingly prevalent in the field of perioperative risk assessment (Huddart et al., 2013). Cardiopulmonary exercise testing (CPET) is the most objective and precise means of assessing physical fitness in surgical candidates and has found utility for the prediction and stratification of surgical risk in various clinical fields including thoracic, vascular and abdominal surgery (Moran et al., 2016; Tew et al., 2018; Goodyear et al., 2013; Brunelli et al., 2009). Current guidelines therefore recommend clinical exercise testing before major surgery (Levett et al., 2018). Nevertheless, although substantial progress is being made in the development of such guidelines for perioperative physicians, these guidelines have not addressed sex-related differences in exercise capacity or other CPET-derived variables (Levett et al., 2018). Amongst the most notable differences between males and females is their body composition and exercise capacity: males generally have less fat tissue, more lean mass and a higher aerobic capacity (Sparling, 1980; Loe et al., 2013). Furthermore, it is known that clinical risk profiles and the response to surgical injury differ between males and females. For example, the incidence of infectious complications, as well as cardiovascular risk profiles, differs between sexes (Foxman, 2002; Oberholzer et al., 2000; Falagas et al., 2007; Appelman et al., 2015). In the specific case of CPET, it has been shown that VO_2_ peak, a measure for maximum oxygen uptake, is lower in females than males in a healthy population, even when matched for age, weight and body mass index (Koch et al., 2009). Furthermore, in cardiovascular disease, it has been shown that these differences in CPET-derived variables are related to differences in prognosis. A reduction of 5 ml/kg/min in the VO_2_ peak from the “classical cut-off value” (14 ml/kg/min) has been proposed for females in order to establish an accurate prognosis in cases of heart failure (Corrà et al., 2013).

Non-sex-specific reference values established by Jones et al. and Hansen et al. have traditionally been used in perioperative practice to assess physical fitness and estimate surgical risk for females and males alike (Jones et al., 1985; Hansen et al., 1984; Balady et al., 2010). More males than females were included in these two studies, with the second including male participants only (Hansen et al., 1984). A recently updated systematic review showed that, in studies establishing CPET reference values in healthy adults, females are underrepresented and account for 38% of the total number of included study participants (Takken et al., 2019). Although an increasing number of hospitals in the UK use CPET to assess surgical risk, separate reference values for males and females have not been reported in recent perioperative CPET publications (Levett et al., 2018; Reeves et al., 2018).

This phenomenon is not unique to the field of perioperative risk assessment: it is seen in a broad range of research fields. More than 25 years after the introduction of the US National Institute of Health Revitalization Act of 1993, which required the enrolment of female participants in federally supported Phase III clinical trials (Labor USCSCo, Resources H, 1993), sex-related differences are disregarded in a large proportion of clinical research (Rochon et al., 1998; Gupta & Wenger, 2012). Epidemiological and clinical studies have often shown differences between males and females in terms of disease incidence, aetiology and response to therapy (Light et al., 2006; Siegel et al., 2017; National Institutes of Health (NIH), 2018). Females are more likely to suffer adverse drug reactions (Patel et al., 2007; Pirmohamed et al., 2004) and a study from 2005 reported that eight out of ten prescription drugs were taken off the market due to health issues in females (Simon, 2005). Despite these issues, both the inclusion of females and female-specific subgroup analyses are underreported in the literature. In the era of precision medicine and personalised treatment, this is a surprising observation with striking and significant implications.

In summary, despite the rapid increase in the prevalence of the clinical application of perioperative CPET, little research is taking place to elucidate differences between the sexes and whether those differences have any bearing on the applicability of CPET in the prediction of surgical risk (Balady et al., 2010). The aim of the present analysis is to highlight this gap in the current literature and to perform a brief analysis of sex-related differences in CPET-derived variables from a recently published multi-centre observational study in patients scheduled to undergo perioperative CPET and major colorectal surgery.

## Methods

We performed a post hoc analysis of a cohort of 703 consecutive patients, of whom 428 were male, in multiple centres in the UK. The design and results of this study have been described elsewhere (West et al., 2016). In short, all patients underwent CPET before major elective surgery in line with American Thoracic Society/American College of Chest Physicians recommendations (Society, 2003). Clinical outcomes including complications, length of stay and mortality were recorded. The primary outcomes of our analysis were oxygen uptake (VO_2_) at anaerobic threshold (AT) and at peak exercise for males and females. Several secondary outcome variables were also assessed, including ventilatory equivalents for carbon dioxide (V_E_/VCO_2_) at AT and heart rate (HR) at peak exercise. Kolmogorov-Smirnov tests were used to assess the normality of the distribution of variables. Variables were reported as mean ± SD or as frequency (percentage). ANOVA was used for normally distributed variables and Bonferroni correction was applied for multiple variable testing. *P* values < 0.05 were considered significant.

In addition, studies investigating the use of CPET for surgical risk assessment and including over one hundred patients were identified and screened for the proportion of female study participants. These studies were selected in a non-systematic way, using expert opinion and lists of studies included in recent review articles. As this is a timeless issue, no studies were excluded on the basis of publication year.

## Results

Table 1 shows the patient characteristics and CPET-derived variables before colorectal surgery analysed separately for males and females. The majority of patients (*n* = 428) were male. In our cohort, there were no significant differences between the groups in terms of age, BMI, tumour stage, laparoscopic approach or clinical outcomes. The primary CPET-derived outcomes, VO_2_ at AT and VO_2_ at peak, were 1.2 L/min and 1.6 L/min, respectively, for males. In females, VO_2_ was 0.8 L/min at AT and 1.1 L/min at peak. The male-female differences were therefore 33% and 25%, respectively. When adjusted for body weight, the differences between males and females were 14% and 18% at AT and peak, respectively. All differences were statistically significant (see Table 1). There was no statistically significant difference between males and females in terms of V_E_/VCO_2_ at AT or heart rate (HR) at peak exercise.

**Table 1:** Patient characteristics, cardiopulmonary exercise testing variables and clinical outcomes of patients undergoing major colorectal surgery

	Males, ***n*** = 428	Females, ***n*** = 275	***P*** value
**Age (years)**	68 ± 11	68 ± 12	0.640
**BMI (kg/m**^**2**^**)**	28 ± 5	28 ± 6	0.653
**TNM**			0.663
0	11 (4.5%)	5 (3.4%)	
1	17 (6.9%)	13 (8.8%)	
2	46 (18.8%)	32 (21.8%)	
3	134 (54.7%)	76 (51.7%)	
4	37 (15.1%)	21 (14.3%)	
**Laparoscopy**			0.407
Yes	169 (39.5%)	100 (36.4%)	
No	259 (60.5%)	175 (63.6%)	
Cardiopulmonary exercise test variables			
**VO**_**2**_**at AT (L/min)**	1.2 ± 0.5	0.8 ± 0.3	0.000
**VO**_**2**_**at AT (mL/kg/min)**	13.1 ± 4.6	11.3 ± 3.9	0.000
**VO**_**2**_**peak (L/min)**	1.6 ± 0.5	1.1 ± 0.3	0.000
**VO**_**2**_**peak (mL/kg/min)**	21.0 ± 6.6	17.2 ± 5.3	0.000
**V**_**E**_**/VCO**_**2**_**AT**	31.1 ± 6.4	30.5 ± 8.1	0.236
**WR peak (watts)**	114.7 ± 45.7	73.0 ± 33.4	0.000
**HR peak (bpm)**	134 ± 21.2	133 ± 22.9	0.805
Clinical outcomes			
**Length of stay (days)**	9.9 ± 9.7	9.6 ± 6.9	0.696
**Complications**			
Yes	279 (65.2%)	166 (60.4%)	
No	149 (34.8%)	109 (39.6%)	
**30-day mortality**	7 (1.6%)	5 (1.8%)	0.855
**1-year mortality**	20 (4.7%)	16 (5.8%)	0.502

*AT* anaerobic threshold, *BMI* body mass index, *bpm* beats per minute, *HR* heart rate, *kg* kilogrammes, *VO*_*2*_ volume of oxygen, *WR* work rate

A total of seventeen studies were screened to determine the numbers of male and female participants. The results can be found in Table 2. Four studies did not report the sex of their participants. In the remaining studies, which included 5117 patients in all, 68.5% of participants were male. Sex-specific analyses of outcome data were performed in two studies (West et al., 2013; Bernal et al., 2014). One study noted that the proportion of females who were classified as high-risk on the basis of oxygen uptake at AT was much larger than the proportion of males. However, no separate outcome analyses were made for both sexes in that study (Wilson et al., 2010).

**Table 2:** Summary of the number of included males and females in recent studies with more than 100 participants that investigated the use of CPET for surgical risk assessment before major surgery

Primary author, year	Target population	Sample size	M/F	% females	Sex-specific analysis
Older, 1993	Patients older than 60 scheduled for major intra-abdominal surgery	*n* = 187	NR	**NR**	No
Older, 1999	Patients scheduled for major intra-abdominal surgery	*n* = 548	NR	**NR**	No
Carlisle, 2007	Patients after repair for unruptured AAA	*n* = 167	NR	**NR**	No
Snowden, 2010	Patients assessed for major surgery with low subjective functional capacity	*n* = 171	107/64	**37.4%**	No
Wilson, 2010	Patients older than 55 assessed for colorectal, bladder, or kidney cancer	*n* = 847	507/340	**40.1%**	No^a^
Ausania, 2012	Patients scheduled for pancreaticoduodenectomy	*n* = 124	67/57	**45.9%**	No
Colson, 2012	Patients scheduled for major thoraco-abdominal surgery	*n* = 1725	1121/604	**35.0%**	No
Hartley, 2012	Patients scheduled for elective AAA repair	*n* = 415	349/66	**15.9%**	No
Prentis, 2012	Patients scheduled for elective AAA repair	*n* = 185	161/24	**12.9%**	No
Lai, 2013	Patients scheduled for colorectal surgery	*n* = 269	NR	**NR**	No
Lee, 2013	Patients scheduled for colorectal surgery	*n* = 112	65/47	**42.0%**	No
Bernal, 2014	Patients scheduled for liver transplant surgery	*n* = 223	151/72	**32.3%**	Yes
Dunne, 2014	Patients scheduled for liver surgery	*n* = 197	138/59	**29.9%**	No
Neviere, 2014	Patients scheduled for liver transplant surgery	*n* = 263	198/65	**24.7%**	No
West, 2014	Patients scheduled for major colonic surgery	*n* = 136	89/47	**34.6%**	Yes
Grant, 2015	Patients scheduled for elective AAA repair	*n* = 506	418/88	**17.4%**	No
Rose, 2018	Patients scheduled for surgical treatment for colorectal cancer	*n* = 213	126/87	**41.0%**	No

^a^The authors do note a male/female difference in the number of patients classified as “unfit” or “high-risk” and suggest that this should be looked at in further research. However, there was no separate analysis of outcome

## Discussion

To our knowledge, this is the first study to report differences between male and female CPET-derived exercise-testing variables in a perioperative setting. VO_2_ at AT and peak are currently the CPET variables most commonly used to assess surgical risk (Moran et al., 2016; Levett et al., 2018). We found remarkable differences for both these variables (male-female differences of 33 and 25%, respectively) in our dataset, even when corrected for body weight (14 and 18%, respectively). Although these observations are not surprising—given the known differences in strength and fitness between males and females—this is the first study to report of such differences in the context of surgical risk assessment.

### Known differences in sex-based physiology

Several factors may contribute to sex-based differences in the assessment of cardiopulmonary fitness in the context of surgical risk assessment. Firstly, and most obviously, male and female body composition is not the same. The differences in the distribution of fat and muscle tissue may be one of the causes of the sex-related difference in physical fitness we observed (Geer & Shen, 2009). However, this is not adequately resolved by correcting for body weight, and studies show that, even when correcting specifically for body fat, there is still a difference in oxygen uptake between the sexes (Sparling, 1980). Secondly, the female body reacts differently to physical stress and stressors related to treatment and disease. Females generally have a lower stroke volume, higher heart rate and lower cardiac output (Hart et al., 2009). When CPET is used for surgical risk stratification, a physical stressor is provoked which increases oxygen demand. Adaptive mechanisms, such as increased heart rate and respiratory rate, are the consequence of sympathetic nervous system activation, increasing oxygen uptake. In males, increasing vascular resistance and therefore blood pressure is the primary response to physical exertion, whereas the main response in females is an increase in heart rate (Hart et al., 2009). It is also known that, at all ages, females have a lower sympathetic drive and lower blood levels of norepinephrine than males, and this is a possible contributory factor to lower maximum oxygen uptake. So, even though current guidelines highlight the need for further research to address the interpretation of perioperative CPET and also discuss the difficulty of interpreting results for obese and cachectic individuals, differences between males and females have not previously been addressed in the perioperative setting (Levett et al., 2018).

### Sex-based differences, CPET and perioperative risk

Oxygen uptake at peak or at AT is used to predict how well patients will adapt to a different stressor in their near future: surgery. Surgery also leads to increased oxygen demand, leading to a number of metabolic and endocrine changes that are collectively known as the surgical stress response.

Although females have a lower maximum oxygen uptake, they do not seem to have a less efficient surgical stress response than males. As seen in our cohort, average outcome after surgical treatment for colorectal cancer seems similar and, in some studies, more in favour of females than of males (West et al., 2016; González et al., 2005; De Angelis et al., 2014). Females seem to adapt to surgical stressors similarly to males, despite having a lower maximum oxygen uptake as a group. When predicting surgical risk, then, the average oxygen uptake capacity of sexes should be taken in account and it would therefore seem illogical to use the same reference values for males and females when assessing their risk of surgical complications and adverse outcome.

In addition, long-known sex-related differences in energy metabolism and physical performance may play a role (Björntorp PAJTAjocn, 1989). During moderate exercise, females primarily utilise fatty acids, preserving muscle glycogen reserves. This enables them to sustain low to moderate exercise for a long time. By contrast, males use glycogen reserves quickly, possibly enabling performance of shorter-lasting exercise with relatively high levels of intensity. An example of such a short, high-intensity, exercise is the steep-ramp protocol, which is mostly used in perioperative CPET (Society, 2003). A different protocol with a stronger focus on moderately intense exercise capacity of longer duration may produce contrasting sex-related differences and merits evaluation for the assessment of oxygen uptake capacity in both female and male surgical candidates.

The observations, and mechanisms, explored above suggest that simply correcting for body weight when applying CPET for surgical risk stratification does not account for the observed difference between sexes. Our assessment of recent studies investigating perioperative fitness using CPET showed that the current pool of research participants in published studies comprises more males (68.5%) than females. Sex-specific analysis is rarely performed in these studies (two out of seventeen studies), and one study even used a completely male reference population to compare with a mixed-sex study population (Rose et al., 2018). There seems to be little awareness of the possibility that not considering the sex of surgical candidates may lead to less effective risk assessment.

In heart failure, where one of the applications of CPET has been the timing of heart transplantation, studies show that the relationship between CPET-derived variables and prognosis is different between males and females, although the same reference values have been used historically for both sexes (Ehrman et al., 2018). Furthermore, a similar inclusion bias is seen in these studies comparable to our findings: more males were included than females and reference values were therefore skewed towards male standards. Accordingly, in addition to inaccurate information about their prognosis, females were given heart transplants too soon, and this may have led to the inefficient use of scarce donor hearts, higher mortality in male patients on waiting lists and higher health care costs. The implication is that assessing female patients on the basis of a predominantly or exclusively male standard can have adverse outcomes for both sexes.

**Fig. 1:**
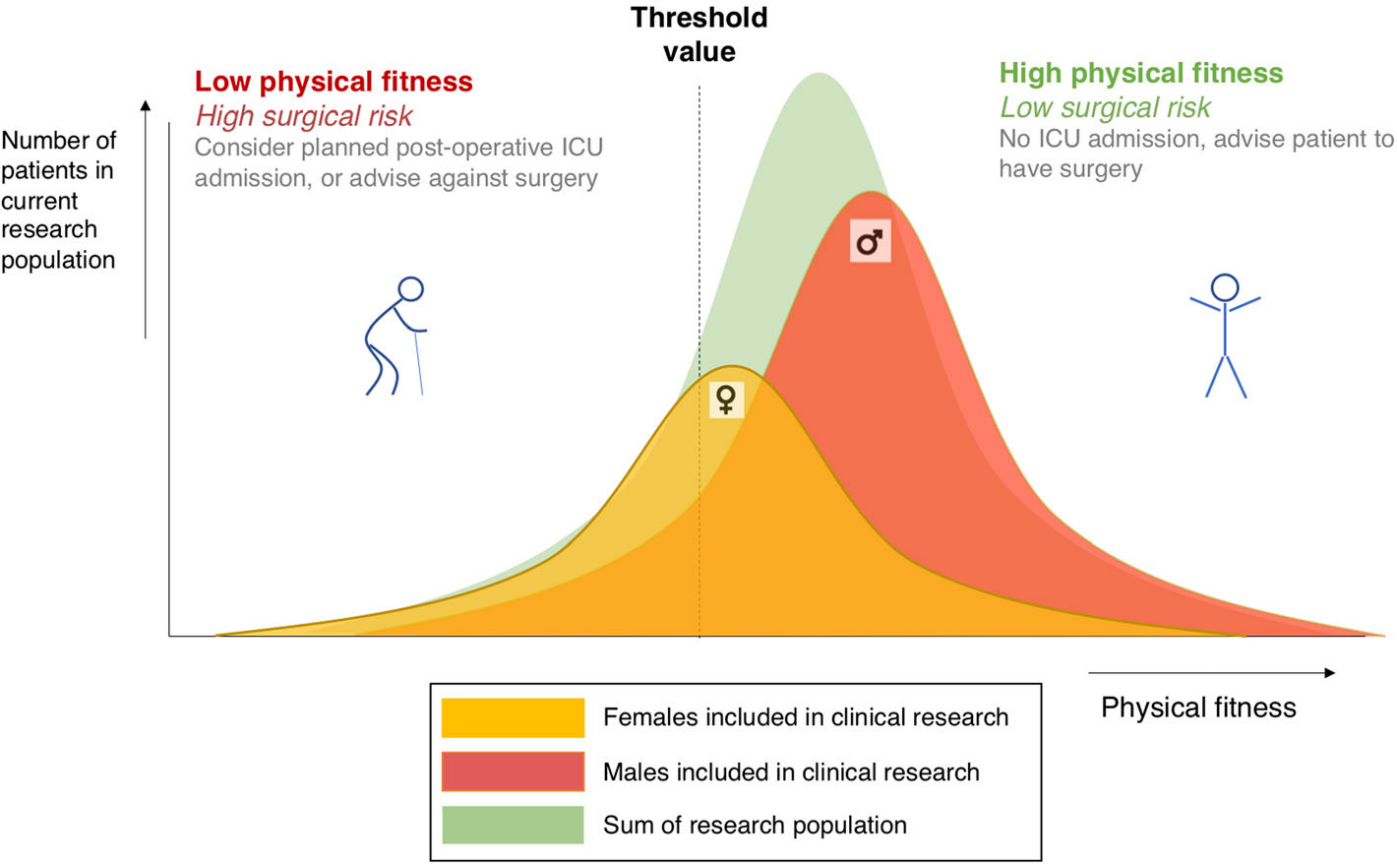
Clinical consequences of applying threshold values from cohorts with mainly male participants to both sexes. This schematic graph presents the distribution of male and female physical fitness, as assessed using CPET parameters such as oxygen uptake at AT. Female distribution is shown on the left in yellow; male distribution is shown on the right in red. The threshold value at which patients are allocated to a “high-surgical-risk population” is based on the total pool of research participants. As shown in the graph, the proportion of females included in the high-risk category is much larger than the proportion of males because there are more males than females in the total study population. AT, anaerobic threshold; CPET, cardiopulmonary exercise testing

Preoperative CPET suffers from similar problems: applying the current risk stratification models—with their lack of discrimination for sex—in clinical decision-making will lead to females being assessed on the basis of male-oriented standards and therefore the overestimation of surgical risk for females (Fig. 1). As a result, females may receive different perioperative interventions or have consultations in which shared decision-making is biased and they may even be advised to forego surgical treatment. Another clinical implication is triage to more intensive post-operative care environments. The misrepresentation of perioperative risk due to sex-specific imbalances could result in the misallocation of level-two post-operative resources, with obvious cost implications as well as the risk of unintended consequences of excess treatment. On the other hand, the surgical risk for males could be underestimated, putting them at a higher risk of surgical complications and emergency ICU admission. However, due to the large numbers of male participants in studies investigating surgical risk assessment, it is not unreasonable to assume that this effect may be less pronounced than the effect described above for female patients. Nevertheless, adequate reference values for both patient groups are required in order to deliver personalised and cost-effective care.

### Future direction

A stronger focus on generating and exploring sex-specific data, which is currently lacking in a large number of scientific fields, will provide researchers with the tools to further elucidate these differences and the mechanisms behind them. Since we are the first to highlight these sex-related differences in the context of perioperative risk stratification, and using a post hoc analysis of observational, non-randomised data, the results of this study should be interpreted with caution and validated in other, larger cohorts. Given the results described here, a heightened awareness of sex-related differences and more research into male and female performance during preoperative exercise testing is urgently needed to enhance the clinical applicability of CPET before major surgery.

## Conclusions and recommendations

Although we recognise the limitations of the results described above, which are observational in nature, we did find a striking difference between the aerobic capacity of male and female surgical candidates in our study, that has previously gone underreported.

It is clear that there are sex differences to be taken into account in the clinical application of CPET, especially given the low number of female research participants in this field at present. To further interrogate the mechanisms and quantification of these differences in perioperative CPET, the authors recommend that a larger, adequately powered study is needed. Our results, however, resonate with a broader, persistent problem in all scientific fields: a lack of sex-specific research and a smaller number of female research participants, skewing results towards male-specific outcomes and subsequent clinical practice. We therefore recommend that researchers in the perioperative field consider these differences in the design and analysis of future clinical studies and undertake adequately powered studies that allow sufficient patient numbers to perform subgroup sex-specific analyses to assess surgical risk. A better understanding of sex-related differences in physical fitness will allow for sex-specific reference values, leading to further refinement and increased accuracy in perioperative risk assessment, truly personalised care and ultimately a better outcome for both sexes.

## Data Availability

Data sharing is not applicable to this article as no datasets were generated or analysed during the current study.

## Abbreviations

AT: Anaerobic threshold
CPET: Cardiopulmonary exercise testing
HR: Heart rate
V_E_/VCO_2_: Ventilatory equivalents for carbon dioxide
VO_2_: Volume of oxygen
